# Data from the ichthyological collection of the Museu Paraense Emílio Goeldi

**DOI:** 10.3897/zookeys.687.11233

**Published:** 2017-08-02

**Authors:** Timóteo Monteiro da Silva, Juliana Corrêa dos Santos, Victor Amazonas Viegas Ferreira, Lorran Alves da Cruz Ramos, Wolmar Benjamin Wosiacki, Marcos Paulo Alves de Sousa

**Affiliations:** 1 Museu Paraense Emílio Goeldi, Av. Perimetral, 1901 - Terra Firme, 66077-830, Belém, Brazil

**Keywords:** Amazon, dataset, ichthyology, occurrence

## Abstract

This dataset contains information on the occurrence of Neotropical fishes (Actinopterygii, Chondrichthyes, Sarcopterygii) collected in South America, mostly from the Brazilian Amazon. The ichthyology collections of the Museu Paraense Emílio Goeldi (MPEG: http://www.museu-goeldi.br/) include specimens collected between 1900 and 2014. The dataset is now available for public consultation on the Global Biodiversity Information Facility portal (http://www.gbif.org/dataset/b0059a3a-5cab-4a08-8d14-d92c23378e43), and through Sistema de Informação sobre a Biodiversidade Brasileira (http://gbif.sibbr.gov.br/explorador/pt/recurso/62).

## Introduction

The Museu Paraense Emílio Goeldi (MPEG), or Goeldi Museum, located in Belém, Pará, Brazil, is a federal research institution within the Brazilian Ministry of Science, Technology and Communication (MCTIC). The Goeldi Museum is the site of the first Amazonian fish collection in Brazil with specimens dating as far back as the end of the nineteenth century.

The ichthyology collections of the Goeldi Museum receive and preserve material evidence, including specimens and associated data and metadata collected in the field, for research and educational purposes. The collections are a source of information and material used by national and international researchers as well as students of two post-graduate programs at MPEG focused on systematics, taxonomy, and biogeography. Due to its wide geographic range and representation of Amazonian fish diversity, over 60 scientific papers have been published over the last ten years based on specimens and types deposited in the Goeldi collections. The MPEG collections are most representative of the Brazilian Amazon, but also contain records of fishes collected in four other neotropical countries (Chile, Colombia, Panama, and Peru). According to Reis (2013), this region has the richest and most diverse fish fauna of the world, with more than 5400 species described. One of the main sources for this aquatic biodiversity is the Amazon basin, with more than 2000 species of freshwater fish, a quarter of all known freshwater species. Of these, 1800 are endemics (Peixoto et al. 2016). One of the greatest conservation challenges in Brazil currently is to harmonize economic development with the sustainable use and preservation of this tremendous aquatic biodiversity (Santos and Santos 2005). Among the principal threats to aquatic environments are hydroelectric dams, which are being built and planned at a growing rate. Although Brazil, Peru, and Bolivia are currently the countries most directly affected by impacts of hydroelectric power plants, other Southern American countries may feel the effects (Fearnside 2015). Large dams reduce fish biodiversity directly and also block the migration pathways of many species, which can be devastating to Neotropical fishes. Dams also cause changes in the dynamic of river nutrients and other biochemical process in deltas, estuaries, and marine-shelf ecosystems (Winemiller et al. 2016). Without effective conservation policies, the ichthyofauna of South America will be increasingly affected over the next few decades (Reis et al. 2016)

Describing new species is the first step in documenting and conserving biodiversity. However even after a new species is first described, it can take years or decades before a more complete understanding of species-level diversity can be apprehended throughout this vast region (Vari and Malabarba 1998). Scientific collections are a crucial source of information for establishing baseline parameters to help measure the ongoing impacts of development on biodiversity (Zaher and Young 2003). When collections data is properly organized, integrated, and made available for the benefit of pertinent studies, it can become a valuable source of information for planning and monitoring public policies, conservation efforts, and natural resource management (Magalhães et al. 2001). Biodiversity information should be available for policy makers and scientists alike. More often than not such information is not easily available for policy makers, thus hindering scientifically based management decisions (Shanmughavel 2007).

The aim of this paper is to describe and synthesize information about Amazon fish biodiversity represented in the Goeldi Museum collections, providing summaries about taxonomic coverage and geographical distribution in order to facilitate rapid and dynamic access to the records present at MPEG. Biodiversity data in open, digital format has the potential to improve the scientific understandings and contribute to conservation policies (Sousa‐Baena 2014).

With these factors in mind, the digitization of the Goeldi fish collections began in 2003, and records were initially inserted into Excel software; in 2009, they were transferred to Specify (SPECIFY SOFTWARE 6). All records have now been computerized, and are available to the scientific community and general public in the Sistema de Informação sobre a Biodiversidade Brasileira (SiBBr 2017) and in Global Biodiversity Information Facility (GBIF 2017)

**Data published through SiBBr and GBIF**: http://www.gbif.org/dataset/3bc27e57-a84d-4e0c-ba0d-9dbba8299674; http://gbif.sibbr.gov.br/explorador/pt/recurso/62

### Project detail

**Project title**: Computerization of the ichthyological collection of the MPEG.

**Personnel**: Timóteo Monteiro da Silva (student), Marcos Paulo Alves de Sousa (head of informatics), Wolmar Benjamin Wosiacki (curator), Juliana Corrêa dos Santos (student), Victor Amazonas Viegas Ferreira (student), Lorran Alves da Cruz Ramos (student).

**Funding**: Ministério da Ciência, Tecnologia, Inovação e Comunicação (MCTIC); Conselho Nacional de Pesquisa (CNPq).

## Taxonomic coverage

General description of taxonomic coverage:

The taxonomic organization of the collection followed Nelson (1994) and Nelson (2006). Currently, higher taxonomic groups are being reorganized according Betancur-R et al. (2013) and Eschmeyer et al. (2016), however the database update is incomplete and ongoing. The ichthyology collection of MPEG includes 260,000 specimens, distributed in 25,874 lots, representing 28 orders, 102 families, 506 genera, and 1710 species. All species in the collection belong to the classes Actinopterygii, Chondrichthyes, and Sarcopterygii. The three most common orders are Characiformes with 600 species in 13,560 lots, Silurifomes with 389 species in 5,290 lots, and Cichlidae with 211 species in 3,437 lots.

Among these are found 263 type specimens of which 33 are holotypes and 227 are paratypes. 261 of the 263 type specimens were collected during the last 15 years.

All type species found in the collection are detailed below:

List of species with holotype and paratype in the collection:

*Acestridium triplax*, *Archolaemus orientalis*, *Aspidoras gabrieli*, *Aspidoras marianae*, *Characidium nana*, *Characidium papachibe*, *Corydoras urucu*, *Cyphocharax aninha*, *Eigenmannia antonioi*, *Eigenmannia desantanai*, *Eigenmannia guairaca*, *Eigenmannia muirapinima*, *Eigenmannia pavulagem*, *Hemigrammus arua*, *Hemigrammus diagonicus*, *Hyphessobrycon montagi*, *Hypomasticus lineomaculatus*, *Hypopygus benoneae*, *Ituglanis ina*, *Stenolicmus ix*, *Tatia caxiuanensis*, *Tetranematichthys barthemi*, *Tometes ancylorhynchus*, *Tometes camunani*, *Tometes kranponhah*, *Trichomycterus guaraquessaba*, *Trichomycterus igobi*, *Trichomycterus mboycy*, *Trichomycterus naipi*, *Trichomycterus papilliferus*, *Trichomycterus plumbeus*, *Trichomycterus taroba*, *Xenurobrycon varii*.

List of species with only paratype in the collection:

*Adontosternarchus duartei*, *Anchoviella juruasanga*, *Ancistrus krenakarore*, *Ancistrus ranunculus*, *Apteronotus lindalvae*, *Apteronotus soneiro*, *Archolaemus ferreirai*, *Archolaemus janeae*, *Archolaemus luciae*, *Archolaemus santosi*, *Aspidoras gabrieli*, *Aspidoras marianae*, *Astroblepus nettoferreirai*, *Baryancistrus chrysolomus*, *Baryancistrus xanthellus*, *Bryconamericus pinnavittatus*, *Centromochlus orca*, *Chaetostoma jegui*, *Crenicichla anamiri*, *Cyphocharax jagunco*, *Cyphocharax lundi*, *Eigenmannia matintaperera*, *Eigenmannia meeki*, *Eigenmannia sayona*, *Eigenmannia waiwai*, *Furcodontichthys novaesi*, *Hassar gabiru*, *Hassar shewellkeimi*, *Hypostomus delimai*, *Hypostomus hoplonites*, *Ituglanis goya*, *Jupiaba citrina*, *Leporinus multimaculatus*, *Moenkhausia celibela*, *Moenkhausia chlorophthalma*, *Moenkhausia eurystaenia*, *Moenkhausia mikia*, *Moenkhausia petymbuaba*, *Moenkhausia plumbea*, *Nemuroglanis furcatus*, *Parotocinclus halbothi*, *Peckoltia compta*, *Peckoltia feldbergae*, *Phallobrycon synarmacanthus*, *Physopyxis ananas*, *Polycentrus jundia*, *Scoloplax baskini*, *Synbranchus lampreia*, *Trichomycterus anhanga*, *Trichomycterus balios*, *Trichomycterus cachiraensis*, *Trichomycterus crassicaudatus*, *Trichomycterus poikilos*, *Trichomycterus trefauti*, *Trichomycterus tupinamba*, *Tyttobrycon marajoara*.

## Taxonomic ranks

**Kingdom**: Animalia

**Phylum**: Chordata

**Classes**: Actinopterygii, Chondrichthyes, Sarcopterygii

**Figure 1.**
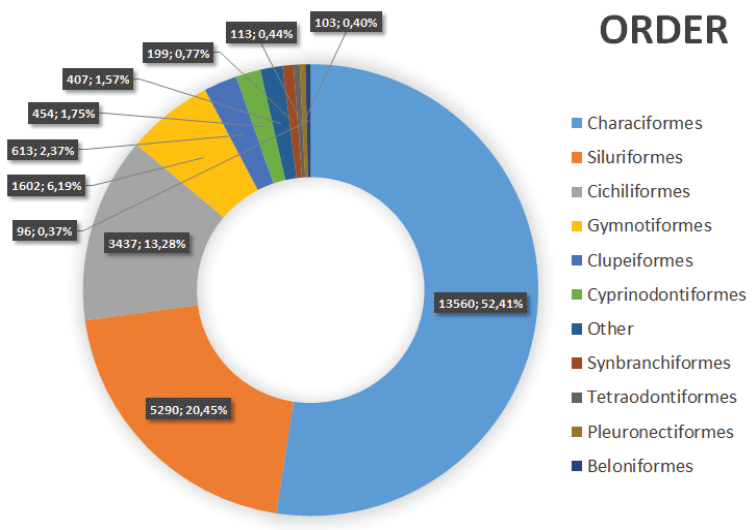
Distribution of species in the MPEG. Number of species and frequencies are represented for each order.

**Orders**: Atheriniformes, Batrachoidiformes, Beloniformes, Carcharhiniformes, Characiformes, Chimaeriformes, Clupeiformes, Cyprinodontiformes, Elopiformes, Gasterosteiformes, Gobiesociformes, Gymnotiformes, Lepidosireniformes, Lophiiformes, Mugiliformes, Myliobatiformes, Osmeriformes, Osteoglossiformes, Cichliformes, Pleuronectiformes, Pristiformes, Rajiformes, Scorpaeniformes, Siluriformes, Squaliformes, Synbranchiformes, Syngnathiformes, Tetraodontiformes (Figure 1).

**Families** Acestrorhynchidae, Achiridae, Anablepidae, Anostomidae, Apteronotidae, Argentinidae, Ariidae, Aspredinidae, Atherinidae, Atherinopsidae, Auchenipteridae, Batrachoididae, Belonidae, Blenniidae, Bothidae, Callichthyidae, Carangidae, Carcharhinidae, Centrarchidae, Centropomidae, Cetopsidae, Chacidae, Characidae, Chilodontidae, Chimaeridae, Cichlidae, Clupeidae, Crenuchidae, Ctenoluciidae, Curimatidae, Cynodontidae, Cynoglossidae, Cyprinodontidae, Dasyatidae, Diodontidae, Doradidae, Echeneidae, Electrophoridae, Eleotridae, Elopidae, Engraulidae, Ephippidae, Erythrinidae, Gasteropelecidae, Gerreidae, Gobiesocidae, Gobiidae, Gymnotidade, Gymnotidae, Gymnuridae, Haemulidae, Helogeneidae, Hemiodontidae, Hemiramphidae, Heptapteridae, Hypopomidae, Lebiasinidae, Lepidosirenidae, Lobotidae, Loricariidae, Lutjanidae, Megalopidae, Mugilidae, Mullidae, Muraenidae, Myliobatidae, Nematogenyidae, Ogcocephalidae, Ophichthidae, Osteoglossidae, Paralichthyidae, Parodontidae, Pimelodidae, Poeciliidae, Polycentridae, Potamotrygonidae, Pristidae, Pristigasteridae, Prochilodontidae, Prystigasteridae, Pseudopimelodidae, Rajidae, Rhamphichthyidae, Rivulidae, Sciaenidae, Scoloplacidae, Scombridae, Serranidae, Serrasalmidae, Sphyraenidae, Sphyrnidae, Squalidae, Sternopygidae, Stromateidae, Synbranchidae, Syngnathidae, Tetraodontidae, Torpedinidae, Triakidae, Trichiuridae, Trichomycteridae, Triglidae.

## Spatial coverage

**Figure 2.**
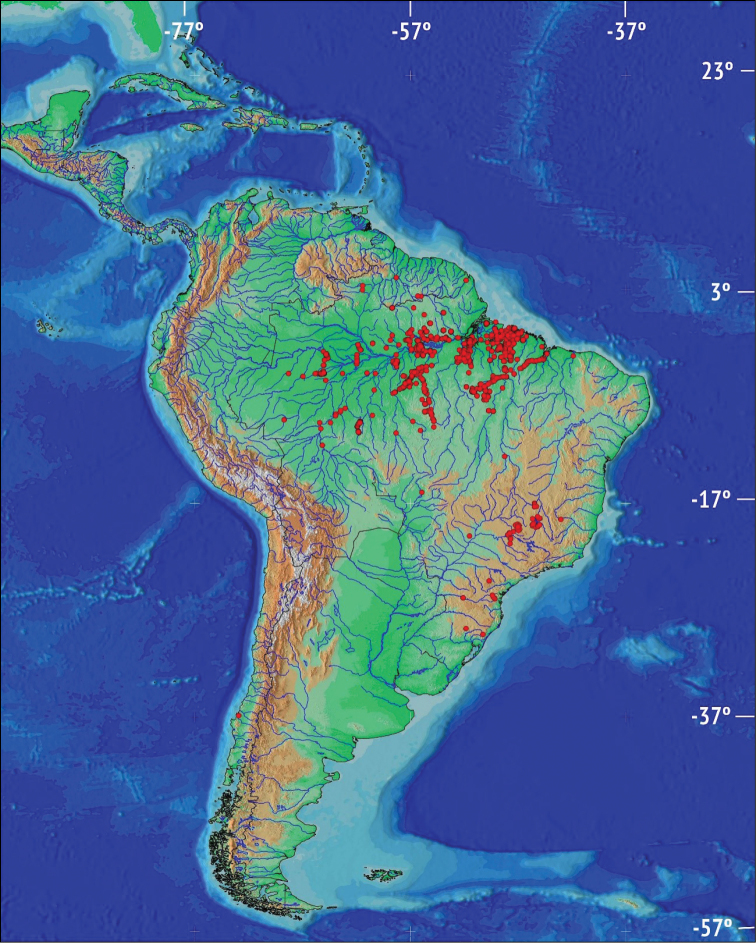
Map of South America showing the localities of all fish specimens with coordinates (dataset available at http://www.gbif.org/dataset/b0059a3a-5cab-4a08-8d14-d92c23378e43). Some dots represent more than one locality.

**General spatial coverage**: The collections include specimens from Brazil, Chile, Colombia, Panama, and Peru. Most samples come from the Brazilian Amazon (Figure 2) from the following river basins: Araguari, Arapiuns, Juruti, Caxiuanã, Madeira, Rio Negro, Teles Pires, Xingu, Amazonas,Guamá, Trombetas, Jamanxim. Other river basins from Brazil represented in the collections include: Parana (Iguaçu, Paranapanema), Tocantins (Anapu, Itacaiunas), and Paraguai (Miranda). The south Atlantic basins is represented by the Laranjeiras river. River basins were attributed used maps from Agência Nacional de Águas (ANA 2017) and the Environmental Ministry (Ministério do Meio Ambiente) of the Brazilian Government.

**Figure 3.**
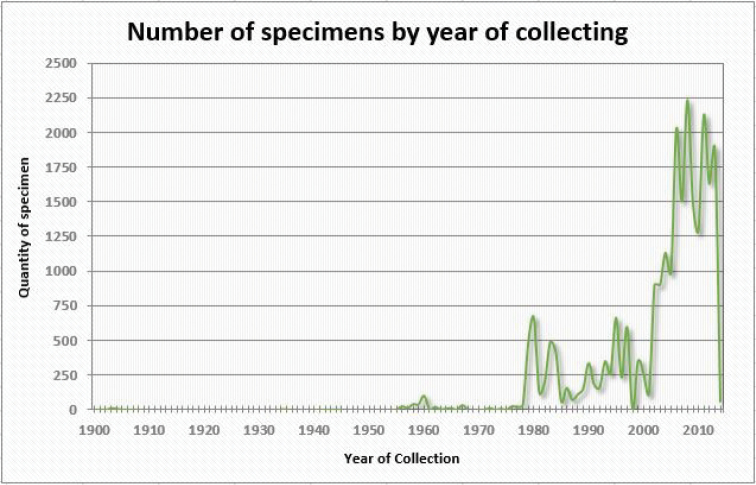
Distribution of the number of specimens collected by year.

**Temporal coverage**: Specimens in the collection date from 1900–2014 (Figure 2) with three significant increments during the early 1980s, in 1995, and after 2000, with more than 600 samples per year. The most recent peak period in collection is observed in 2002-2013, with more than 900 samples per year (Figure 3).

### Natural collections description

**Parent collection identifier**: Museu Paraense Emílio Goeldi

**Collection name**: Ichthyology

**Collection identifier**: MPEG.ICT

**Specimen preservation method**: Alcohol

## Methods

**Figure 4.**
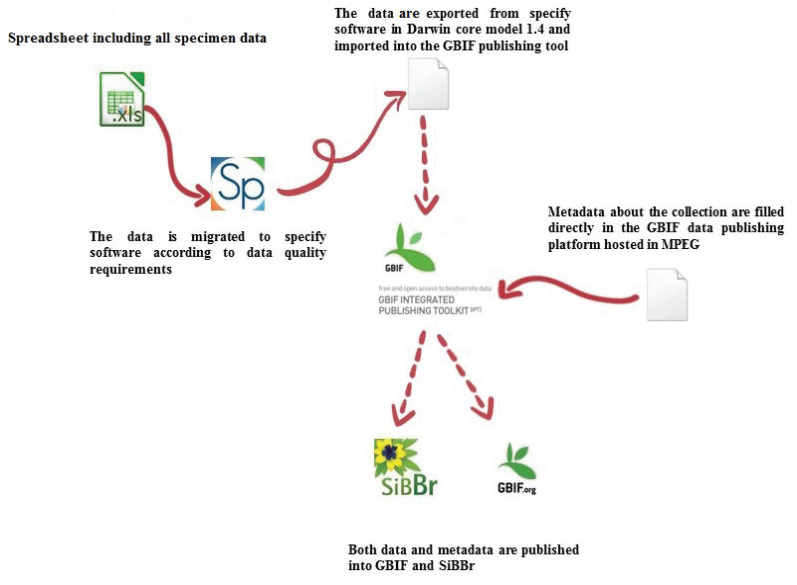
Flow chart of data publication process. Produced by the author.

**General method of publishing**: Samples were obtained from collecting licenses, exchange, donation, or purchase. Samples are stored and preserved in the collection and data is stored in the Biodiversity Data Management System. The main data from specimens that are incorporated in the collection are published in "Sistema de Informação sobre a Biodiversidade Brasileira (SiBBr)" and "Global Biodiversity Information Facility" (GBIF) using an export tool from Specify Software and "Integrated Publishing Toolkit" (IPT) from GBIF which uses the Darwin core Standard version 1.4. The data was imported and published as per the schematic illustration below (Figure 4).

**Sampling description**: During its 150 years of history, the ichthyology collection of MPEG has received collections from dozens of scientists who have used various methods including gillnets, drag and throw (cast) nets, *matapis*, dip nets, sieves, harpoons, snorkeling, diving, etc.

**Quality control description**: The most recent taxonomic organization of the collection followed Nelson (1994), and currently Nelson (2006). Currently, the system is being updated according Betancur-R (2013) and Eschmeyer et al. (2016). Therefore, for purposes of this paper, the definition of large groups still follows Nelson (2006), such that representative groups of the collection, for example, Cichlidae do not belong to Cichliformes. The identification of genus and species still follows the bibliography in Eschmeyeret et al. (2016), but all the data will be updated to Betancur-R et al. (2013).

## Datasets

### Curatorship and storage

The curatorial protocol involves receiving material that is identified and labelled, while data and metadata are digitized and deposited in a two story collection room measuring 192 m^2^, air-conditioned to 22°C. The specimens are fixed in formalin for 50 hours and transferred into a 70% ethanol solution for permanent storage.

The process for the preservation of bone and cartilage samples is based on Taylor and Van Dyke (1985). The samples are stored in glass jars or other kinds of containers (e.g., high-density polyethylene drums) and the collection is organized taxonomically by order and family. Within the families, the genera and species are arranged in alphabetical order. The type material (holotypes and paratypes) is stored in metal cabinets. Protocol for loan, exchange, donation, and collection visits begins with e-mail contact with the curator, who evaluates the proposal and, if needed, requests the curatorial staff to prepare the requested specimens for viewing or shipping to any country.

## Dataset description

**Object name**: Darwin Core Archive Museu Paraense Emílio Goeldi - ichthyology collection

**Character encoding**: UTF-8

**Format name**: Darwin Core Archive format

**Format version**: 11.2

**Distribution**: http://ipt.museu-goeldi.br/ipt/resource?r=museu_paraense_emilio_goeldi_ictiology_collectionand;

http://www.gbif.org/dataset/3bc27e57-a84d-4e0c-ba0d-9dbba8299674

**Publication date of data**: 2015-01-21

**Language**: Portuguese

**Licenses of use**: This dataset is licensed under a Creative Commons Attribution Non Commercial (CC-BY-NC) 4.0 License (https://creativecommons.org/licenses/by-nc/4.0/legalcode).

**Metadata language**: English

**Date of metadata creation**: 2014-08-01

**Hierarchy level**: Dataset
